# Subcritical Hopf Bifurcation and Stochastic Resonance of Electrical Activities in Neuron under Electromagnetic Induction

**DOI:** 10.3389/fncom.2018.00006

**Published:** 2018-02-06

**Authors:** Yu-Xuan Fu, Yan-Mei Kang, Yong Xie

**Affiliations:** ^1^School of Mathematics and Statistics, Xi'an Jiaotong University, Xi'an, China; ^2^School of Aerospace Engineering, Xi'an Jiaotong University, Xi'an, China; ^3^The State Key Laboratory of Strength and Vibration for Mechanical Structures, Xi'an, China

**Keywords:** electromagnetic induction, subcritical Hopf bifurcation, stochastic resonance, weak signal detection, improved FitzHugh-Nagumo model

## Abstract

The FitzHugh–Nagumo model is improved to consider the effect of the electromagnetic induction on single neuron. On the basis of investigating the Hopf bifurcation behavior of the improved model, stochastic resonance in the stochastic version is captured near the bifurcation point. It is revealed that a weak harmonic oscillation in the electromagnetic disturbance can be amplified through stochastic resonance, and it is the cooperative effect of random transition between the resting state and the large amplitude oscillating state that results in the resonant phenomenon. Using the noise dependence of the mean of interburst intervals, we essentially suggest a biologically feasible clue for detecting weak signal by means of neuron model with subcritical Hopf bifurcation. These observations should be helpful in understanding the influence of the magnetic field to neural electrical activity.

## Introduction

Memristor or memory resistor was supposed by Chua (1971) 46 years ago as the forth fundamental circuit device along with resistor, inductor and capacitor, and it was successfully realized by Stan William's group at HP Labs in 2008 for the first time (Strukov et al., 2008). Because of the huge storage potential and the complex nonlinearity, the memristor has recently attracted considerable attention in theoretical and applied neuroscience (Itoh and Chua, 2008; Yogesh and Stephen, 2009; Wen et al., 2013; Bao et al., 2015; Chen et al., 2017; Zha et al., 2017).

In modern society, human being or animals are inevitably more or less exposed in the electrical hazards of ubiquitous electromagnetic radiation, and the fact that this radiation can have severe consequence on biological rhythm and recognition has attracted much attention (World Health Organization, 2011), but how the electromagnetic radiation changes the behavior of neural systems is still unclear. Fortunately, the device of memristor emerges and it can act as a feasible tool for exploring the influence of electromagnetic radiation on neural system activities, since one can keep the consistency of physical dimension (or unit) when modeling the membrane potential and magnetic flux into coupling systems (Wu et al., 2016). Several investigations have been done in this regard. For example, Lv et al. (Lv and Ma, 2016) proposed a comprehensive modified Hindmarsh-Rose neuron model by introducing the magnetic flux as a fourth variable, and Lu et al. (2017) imposed different types of electrical stimulus impended with a high-low frequency current on this improved HR model to investigate mode selection in neural activity. Guo et al. (Ren et al., 2017) used memristor to discuss the polarization and magnetization in excitable neural model. Ma et al. (Wu et al., 2016; Ma et al., 2017) adopted a magnetic flux across the membrane to describe the electromagnetic induction and the spiral waves have been induced. Although the abundant firing patterns have been revealed, the underlying dynamical mechanism responsible for these patterns in these newly-built models has not been disclosed.

In nervous systems, noise not only has various origins but seldom acts as a trivial disturbance (Tanabe and Pakdaman, 2001; Hasegawa, 2004; Faisal et al., 2008; Shao and Kang, 2014; Sun and Shi, 2014). One of the anti-intuitive phenomena of noise is often termed as stochastic resonance (SR), where a suitable noise can amplify the external weak coherent signal under certain nonlinearity. In the absence of electromagnetic interference, many theoretical or experimental literatures have shown that living organisms can utilize noise as a benefit in detecting or transferring weak signal on both cellular and system levels (Mark et al., 2009). Kang et al. (2005) showed the existence of signal-to-noise ratio gain of SR based on the leaky integrate-and-fire neuron model, Jiao and Wang (2010) observed SR under the effect of synaptic transmission noise; Sun and Li (2016) demonstrated that the partial time delay can induce a stochastic multi-resonance in a Watts-Strogatz neuronal network. Nevertheless, to our knowledge the phenomenon of SR has not been explored in neural system under electromagnetic disturbance. Therefore, we naturally wonder whether a weak coherent oscillation in the electromagnetic disturbance can be amplified through SR.

After a modified FitzHugh–Nagumo (FHN) model with flux-controlled memristor is introduced in Section A modified FitzHugh–Nagumo neuron model, some analytical and numerical results on the bifurcation behavior of the system are derived in Section Analysis of bifurcation. And then, SR in the modified model with a weak periodic modulation is exhibited and explained in Section Stochastic resonance. Finally, conclusion and discussion are given in Section Conclusion and Discussion.

## A modified FitzHugh–Nagumo neuron model

Let us start with the conventional FHN neuron model (Fitzhugh, 1961; Nagumo et al., 1962).

(1) $$\{\begin{array}{l} \dot{v}=v(v-a)(1-v)-w+I_{ext}\\ \dot{w}=\varepsilon (v-dw) \end{array}$$

where the fast-varying trans-membrane potential *v* and the slow current variable *w* are treated as dimensionless. In the model (1), the nonlinear term *v*(*v* − *a*)(1 − *v*) stands for total trans-membrane ionic currents per unit area, *I*_*ext*_ is the external forcing current. Since our purpose is to investigate the influence of electromagnetic induction, the external forcing current is set to zero. The parameters ε = 0.02, *d* = 1 and *a* = 0.5 are fixed such that the dynamical evolution of the model (1) starting from any initial state can asymptotically approach a resting equilibrium state when *I*_*ext*_ = 0. We keep this zero external input throughout the context.

In order to consider the effect of electromagnetic induction on membrane potentials of neuron, with the help of Strukov et al. (2008) we employ the memristor to realize the coupling and modulation on membrane potential from magnetic flux for maintaining the consistency of physical meaning (Wu et al., 2016). Note that the memristor characterizes the relation between charge and magnetic flux, so if let *q* be charge and φ the magnetic flux, then a voltage across a charge-controlled memristor can be modeled as *v*(*t*) = *M*(*q*(*t*))*i*(*t*) with $M\left(q\right)=\frac{d\varphi \left(q\right)}{dq}$. In reverse, a flux-controlled memristor should be described by *i*(*t*) = *W*(φ(*t*))*v*(*t*) with $W\left(\varphi \right)=\frac{dq\left(\varphi \right)}{d\varphi }$. Here the explicit forms of *M*(*q*) and *W*(φ) should depend on the design of the device of memristor.

We take a flux-controlled memristor of $W\left(\varphi \right)=\frac{dq\left(\varphi \right)}{d\varphi }=k\left(\alpha +3\beta \varphi^{2}\right)$ (Itoh and Chua, 2008; Bao et al., 2010a; Wu et al., 2016) to modify the conventional FHN model. We choose α = 0.1 and β = 0.02 to generate complex dynamical behavior (Bao et al., 2010a,b; Wu et al., 2016). Thus, the time evolution equation of the membrane potential *v* becomes

$$\dot{v}=v(v-a)(1-v)-w+k(\alpha +3\beta \varphi^{2})v.$$

According to the Faraday's law, the change of the magnetic flux φ is dominated by varying voltage and magnetic flux, we can suppose that the time derivative of φ is a linear function of *v* and φ, that is to say,

$$\dot{\varphi }=k_{1}v-k_{2}\varphi +\varphi_{ext}$$

where φ_*ext*_, as a bifurcation parameter, is used to describe the bias in external forcing magnetic field.

Therefore, the improved FHN model, which takes the effect of electromagnetic induction into consideration, has the following form of a set of three-variable nonlinear ordinary differential equations.

(2) $$\{\begin{array}{l} \dot{v}=v(v-a)(1-v)-w+k(\alpha +3\beta \varphi^{2})v\\ \dot{w}=\varepsilon (v-dw)\\ \dot{\varphi }=k_{1}v-k_{2}\varphi +\varphi_{ext}. \end{array}$$

Although the original FNH model has an exclusively stable asymptotic state for the given parameters of ε, *d*, and *a*, the introduction of electromagnetic induction can induce complex periodic or bursting firing patterns in the modified model. We will explore the involving dynamical mechanism in the absence of noise and in the presence of noise in Sections Analysis of Bifurcation and Stochastic Resonance, respectively.

## Analysis of bifurcation

The concept of bifurcation in nonlinear dynamical theory can be categorized into static bifurcation and dynamic bifurcation. Usually, the former means the change in number or the stability of equilibrium points, while the latter refers to the similar changes relating to limit cycles (Zhang, 2005; Xie et al., 2008a,b). If an equilibrium state is stable, the affiliating system will evolve closer and closer to it if the initial state falls within its basin of attraction; otherwise, the system will leave it forever if the initial state is not exact on it. In neuron model, the stable equilibrium usually stands for a resting state, and the limit cycle corresponds to the repetitive firing state. Bifurcation has been scrutinized analytically and numerically by many researchers in computational neuronal science. Quantities of literatures have dedicated to the bifurcation behavior of neuron models during the past decades (Hassard, 1978; Guckenheimer and Labourian, 1993; Eugene, 2000; Xie et al., 2008b; Jia and Gu, 2017), but the involving investigations have been extended to the improved models with electromagnetic induction, even if the abundant firing patterns have been revealed (Lv and Ma, 2016; Wu et al., 2016; Lu et al., 2017). In this section, we aim to disclose the underlying dynamical mechanism responsible for the emergence of the firing pattern in the improved system (2).

At first, let us pick out the constant solution, namely the equilibrium points of the system (2), where the variables *v*, *w* and φ stay there forever if there is no external perturbation. Suppose *E*_0_(*v*_0_, *w*_0_, φ_0_) is one of the equilibrium points, then there holds

$$\{\begin{array}{l} v_{0}(v_{0}-a)(1-v_{0})-w_{0}+k(\alpha +3{\beta \varphi_{0}}^{2})v_{0}=0\\ v_{0}-dw_{0}=0\\ k_{1}v_{0}-k_{2}\varphi_{0}+\varphi_{ext}=0 \end{array}$$

and thus

$$v_{0}\left[(v_{0}-a)(1-v_{0})-\frac{1}{d}+k\left(\alpha +3\beta {\left(\frac{k_{1}v_{0}+\varphi_{ext}}{k_{2}}\right)}^{2}\right)\right]=0$$

with three solutions given by

$$v_{01}=0,v_{02}=\frac{-B+\sqrt{B^{2}-4AC}}{2A},v_{03}=\frac{-B-\sqrt{B^{2}-4AC}}{2A}$$

where $A=\frac{3kk_{1}^{2}\beta }{k_{2}^{2}}-1\;B=\frac{6kk_{1}\beta \varphi_{ext}}{k_{2}^{2}}+1+a\;C=\;\frac{3k\beta \varphi_{ext}^{2}}{k_{2}^{2}}-a-\frac{1}{d}+k\alpha$. Let *E*_01_, *E*_02_ and *E*_03_ to be the resultant equilibrium points corresponding to *v*_01_, *v*_02_ and *v*_03_, then $E_{01}={\left(0,0,\varphi_{ext}/k_{2}\right)}^{T}$.

**Figure 1:**
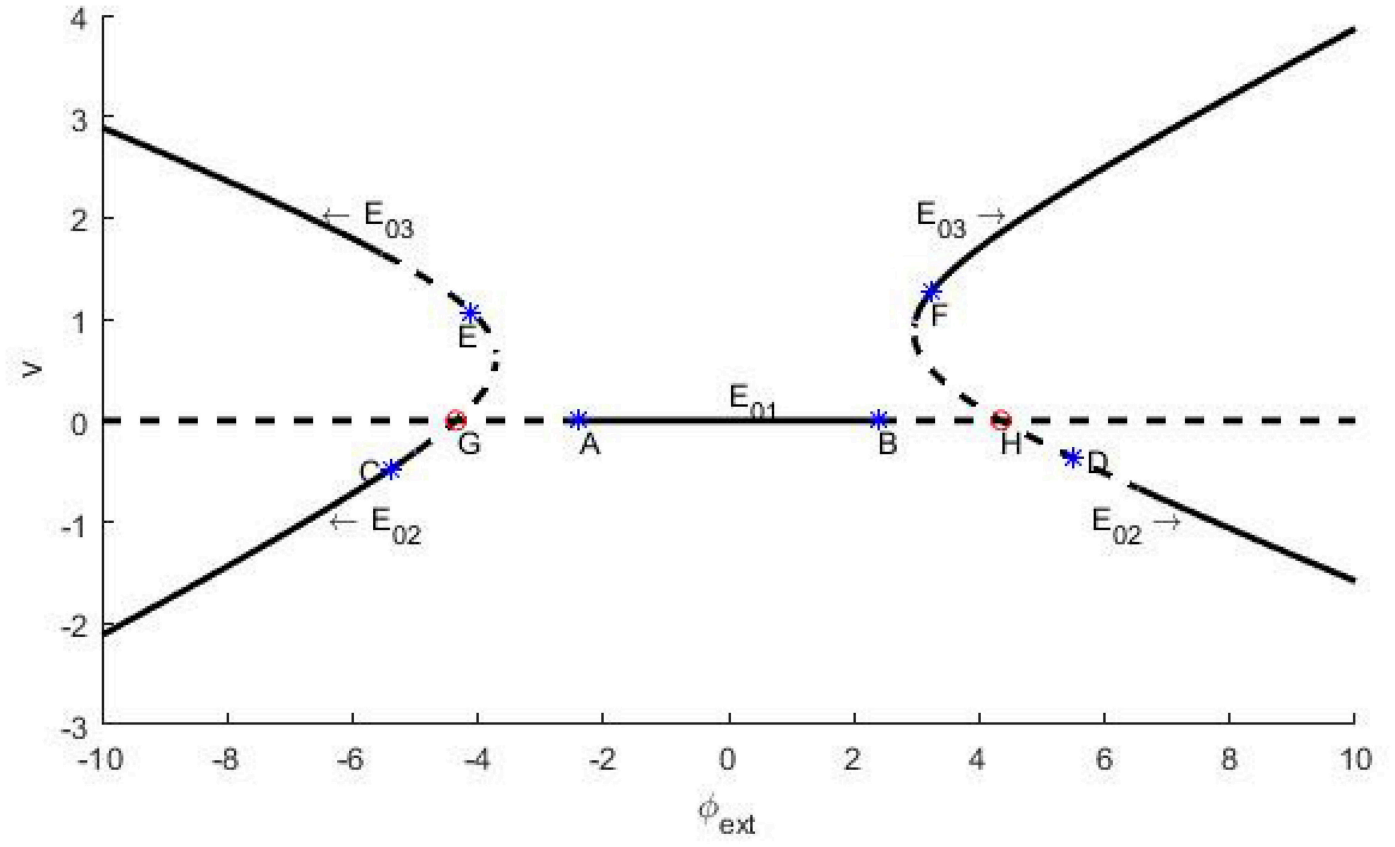
Schema of the branches of equilibrium points for trans-membrane potential and their stability vs. the bifurcation parameter φ_*ext*_. The parameters are set as *k* = 1, *k*_1_ = 0.5, and *k*_2_ = 0.9. The branches locate at lower left and right stand for E_02_ and the branches situate in upper left and right are E_03_. The flat line is E_01_. In figures, the solid curves stand for stable branches, and the dash curves denote unstable branches. Remark: The figure is plotted by the software of xppaut.

Next, let us explore the stability of the three equilibrium points with φ_*ext*_ as bifurcation parameter. Note that the stability of an equilibrium point is determined by the eigenvalues of its Jacob matrix. That is to say, if all the eigenvalues are of negative real part, then the equilibrium point is stable, otherwise it might be marginally stable or unstable. According to the distinction criteria (Zhang, 2005), branches of the equilibrium points and their stability on the φ_*ext*_ − *v* plane are shown in Figure 1, where the solid curves represent the stable branches, the dash lines indicate the unstable ones, and evidently the points A~H indicate the occurrence of bifurcation.

In fact, we can further distinguish the bifurcation types of the bifurcation points A~H. In general, the appearance of a pair of pure imaginary eigenvalues signifies Hopf bifurcation, and the emergence of a zero eigenvalue predicates fork-type or saddle-node bifurcation (Zhang, 2005). In this paper, our emphasis is put on the identification of Hopf bifurcation. For the system (2), if the resultant 3 × 3 Jacobi matrix has one eigenvalue of negative real part and two zero real parts at some critical value of the bifurcation parameter, then as usual we say that Hopf bifurcation occurs, through which a constant membrane potential solution becomes unstable, but a stable periodically oscillatory action potential solution appears.

Let us take the equilibrium point *E*_01_ as example to demonstrate how Hopf bifurcation occurs. From the linearization Jacobian matrix of the system (2)

$$J(v,w,\varphi )=\left[\begin{array}{ccc} -3v^{2}+2(1+a)v-a+k(\alpha +3\beta \varphi^{2}) & -1 & 6k\beta \varphi v\\ \varepsilon & -\varepsilon d & 0\\ k_{1} & 0 & -k_{2} \end{array}\right],$$

One can easily obtain the characteristic determinant of system (2) at *E*_01_ as

$$\begin{array}{l} |\lambda I_{3}-J(0,0,\frac{\varphi_{ext}}{k_{2}})|\;=\left|\begin{array}{ccc} \lambda -A_{1} & 1 & 0\\ -\varepsilon & \lambda +\varepsilon d & 0\\ -k_{1} & 0 & \lambda +k_{2} \end{array}\right|\\ \;=(\lambda +k_{2})[(\lambda -A_{1})(\lambda +\varepsilon d)+\varepsilon ]=0 \end{array}$$

with $A_{1}=-a+k\left(\alpha +3\beta {\left(\frac{\varphi_{ext}}{k_{2}}\right)}^{2}\right)$. Obviously, there is an eigenvalue λ = −*k*_2_ satisfying Re(*k*_2_) ≠ 0, and thus if a pair of pure imaginary eigenvalues λ = ±*iω* exists, there must hold true

$$-\omega^{2}\pm (\varepsilon d-A_{1})\omega \;i-A_{1}\varepsilon d+\varepsilon =0$$

i.e.,

$$\{\begin{array}{l} -\omega^{2}-A_{1}\varepsilon d+\varepsilon =0\\ \varepsilon d-A_{1}=0, \end{array}$$

which leads to *A*_1_ = ε*d* and $\omega =\sqrt{\varepsilon -\varepsilon^{2}d^{2}}$. Thus, the branch of the equilibrium point *E*_01_ undergoes two Hopf bifurcations at parameter

$$\varphi_{ext}=\pm \sqrt{\frac{\varepsilon d+a-k\alpha }{3k\beta }}k_{2}$$

which can be reduced to $\varphi_{ext}^{\left(1\right)}=-2.381$ and $\varphi_{ext}^{\left(2\right)}=2.381$ for given parameters as denoted in the caption of Figure 1. Clearly, they correspond to the bifurcation points *A* and *B* on Figure 1.

Similar to the above analysis, we also can know that the branch of the equilibrium point *E*_02_ undergoes Hopf bifurcations at *C* and *D*, and the branch of the equilibrium point *E*_03_ undergoes Hopf bifurcations at points *E* and *F*, with the bifurcation parameter φ_*ext*_ being −5.386(*C*), 5.512(*D*), −4.113(*E*) and 3.236(*F*), respectively. Further, we can confirm but skip the details that *G* and *H*, as the intersections of the branches of *E*_01_ and *E*_02_, are saddle-knot bifurcation points with bifurcation parameters φ_*ext*_ = ±4.347, which essentially. Here we stress that all of the numerical errors do not exceed 0.001.

**Figure 2:**
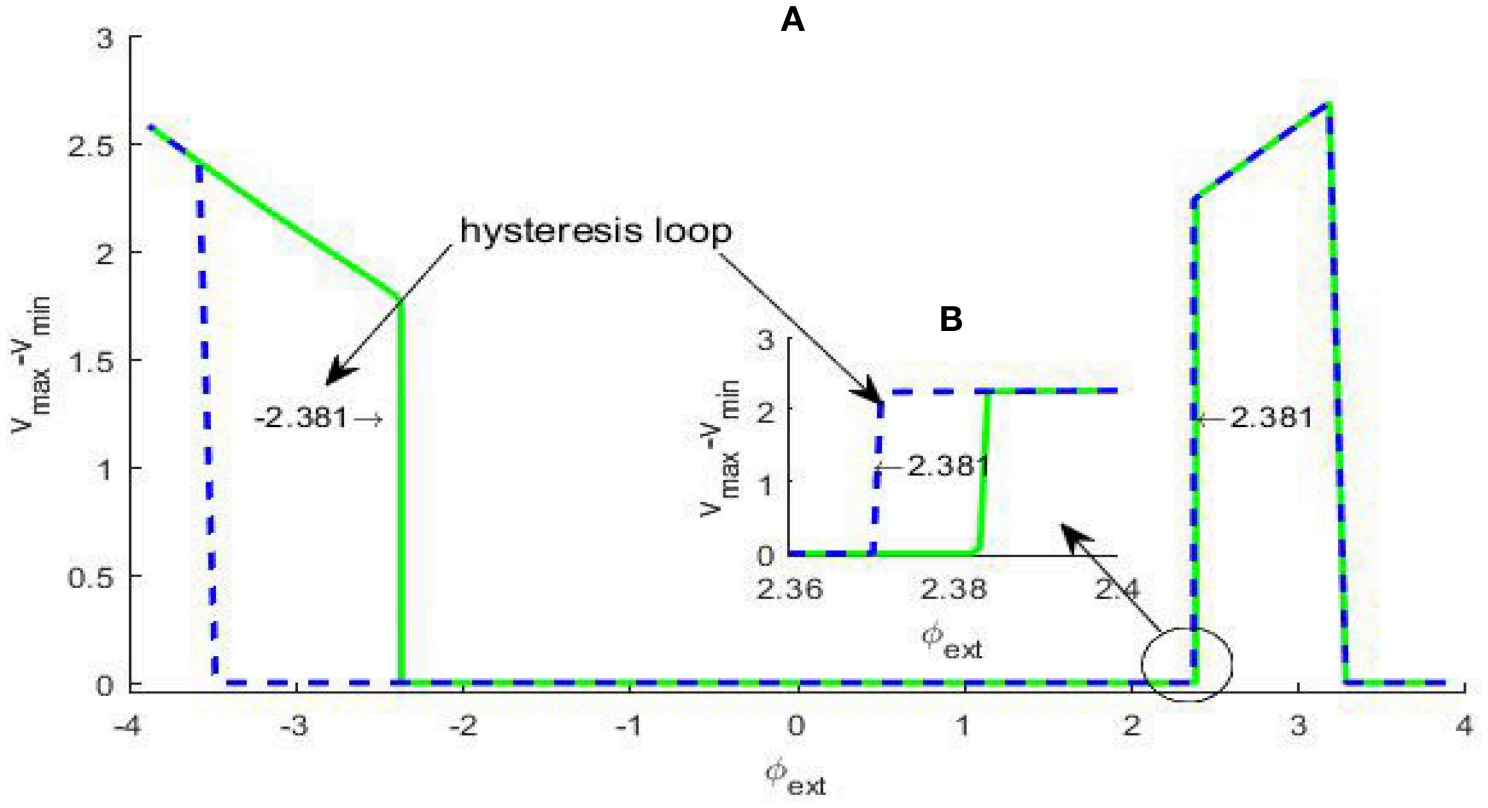
Dependence of the trans-membrane potential difference on the bifurcation parameter φ_*ext*_ **(A)**. The green solid branch shows how the trans-membrane potential difference changes as the parameter φ_*ext*_ increases, while the blue dash branch gives the change trend as the parameter φ_*ext*_ decreases. We can see that within certain parameter range there are two hysteresis loops, the left-hand one is evident and the magnification of the right-hand one **(B)** has been inserted in the figure. The existence of the two hysteresis loops show the bifurcation at ±2.381 both are subcritical Hopf bifurcation.

The occurrence of Hopf bifurcation has been verified by the existence of a pair of pure imaginary eigenvalues, but Hopf bifurcation can further distinguished into two types: subcritical Hopf bifurcation and supercritical Hopf bifurcation by checking dependence of the membrane potential difference over a sufficiently large time span via bifurcation parameter. In general, in subcritical Hopf bifurcation the oscillatory solution occurs before the bifurcation point, but in supercritical Hopf bifurcation the oscillatory solution emerges after the bifurcation point, and whether a hysteresis loop exists is a typical distinction criteria. In fact, if for some given parameter (*v*_max_ − *v*_min_) ≈ 0, we can regard that the system approaches an equilibrium state; otherwise, we can pick out an oscillatory solution, which can correspond to the limit cycle in the phase space if the occurrence of the oscillation is due to a bifurcation induced by an equilibrium point. For the system (2), the difference (*v*_max_ − *v*_min_) vs. the varying φ_*ext*_ is depicted in Figure 2. From the picture, a hysteresis loop can be clearly observed near $\varphi_{ext}^{\left(1\right)}$, and another hysteresis loop can be captured near $\varphi_{ext}^{\left(2\right)}$ by some partial amplification., therefore the Hopf bifurcations at *A* and *B* are both subcritical.

**Figure 3:**
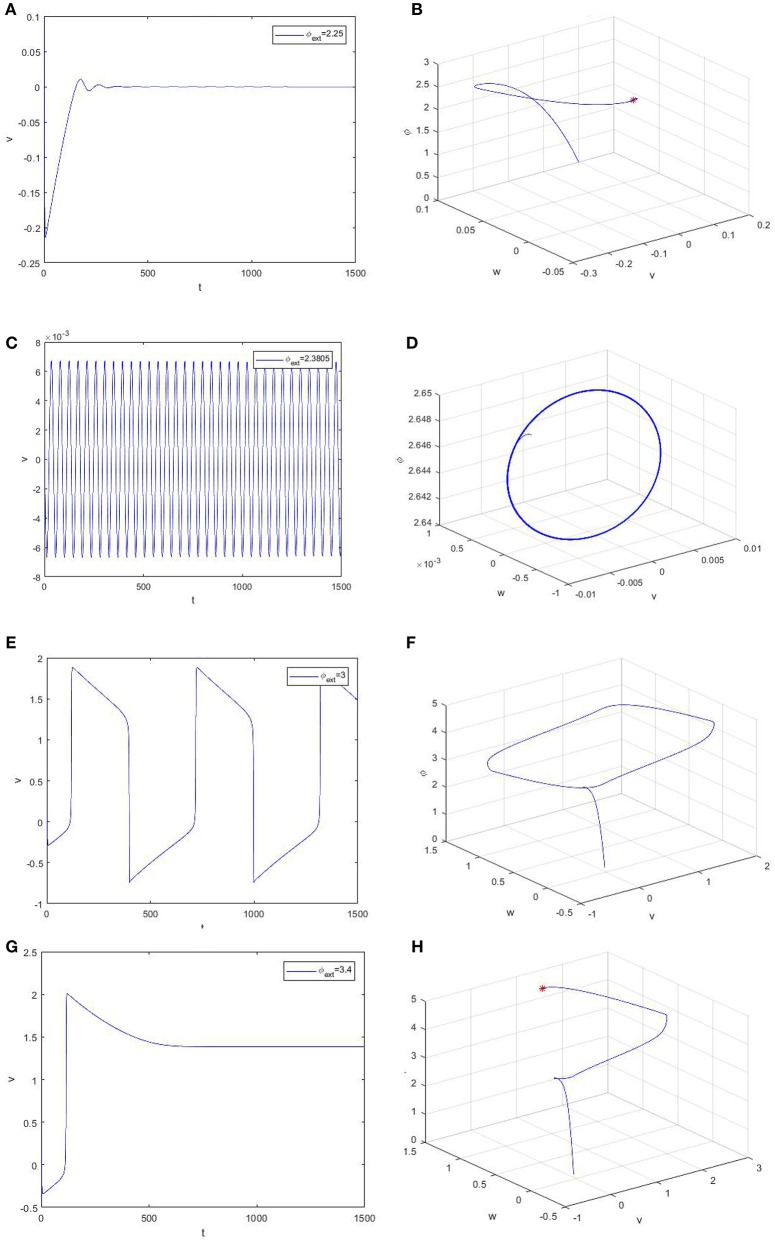
Time evolution of the trans-membrane potential and the phase diagram of the model (2). From up to down, the bifurcation parameter φ_*ext*_ is equal to 2.25 **(A,B)**, 2.3805 **(C,D)**, 3.0 **(E,F)**, and 3.4 **(G,H)**, respectively.

For an intuitive understanding, we depict the time series of the trans-membrane potential and the phase diagram when φ_*ext*_ > 0 in Figure 3. As Figure 3 shows, the membrane potential will stay at the resting level when the bifurcation parameter is less than the critical bifurcation (Figures 3A,B), but it will evolve according to a periodic motion as the bifurcation parameter increases (Figures 3C–F). More precisely, the Figures 3C–F respectively correspond to subthreshold oscillation and superthreshold oscillation (impulsive discharge) of neurons. Moreover, Figures 3G,H reveal that the model (2) will attain another equilibrium state of a high asymptotical membrane potential, which should be morbid for neuronal activity.

## Stochastic resonance

It has been extensively proven that weak periodic signal can be amplified by random fluctuation in many nonlinear systems through the principle of SR (Heneghan et al., 1996). This phenomenon has also been well-documented in neural systems in the absence of electromagnetic induction (André, 1993; Kang et al., 2005; Gosak et al., 2007). Here what we interest in is to check whether a weak subthreshold oscillation in the electromagnetic field can be amplified by a suitable amount of fluctuation.

Assume that the external forcing magnetic field consists of a subthreshold signal and noise, i.e., the form of φ_*ext*_ is described as follows

$$\varphi_{ext}=r\;\sin(2\pi ft)+\varphi_{ext}^{0}+\xi (t)$$

where $r\sin\left(2\pi ft\right)+\varphi_{ext}^{0}$ is a subthreshold signal of bias $\varphi_{ext}^{0}$, that is to say, the particle will not jump from one state to the other if it is only driven by this signal, and ξ(*t*) is Gaussian white noise satisfying < ξ(*t*)) > = 0 and < ξ(*t*)ξ (*s*) > = 2*Dδ*(*t* − *s*). With these factors taken into account, the system (2) can be rewritten into

(3) $$\{\begin{array}{l} \dot{v}=v(v-a)(1-v)-w+k(\alpha +3\beta \varphi^{2})v\\ \dot{w}=\varepsilon (v-dw)\\ \dot{\varphi }=k_{1}v-k_{2}\varphi +r\;\sin(2\pi ft)+\varphi_{ext}^{0}+\xi (t) \end{array}$$

In this section, the same parameters as in Figure 1 are used, unless otherwise stated.

**Figure 4:**
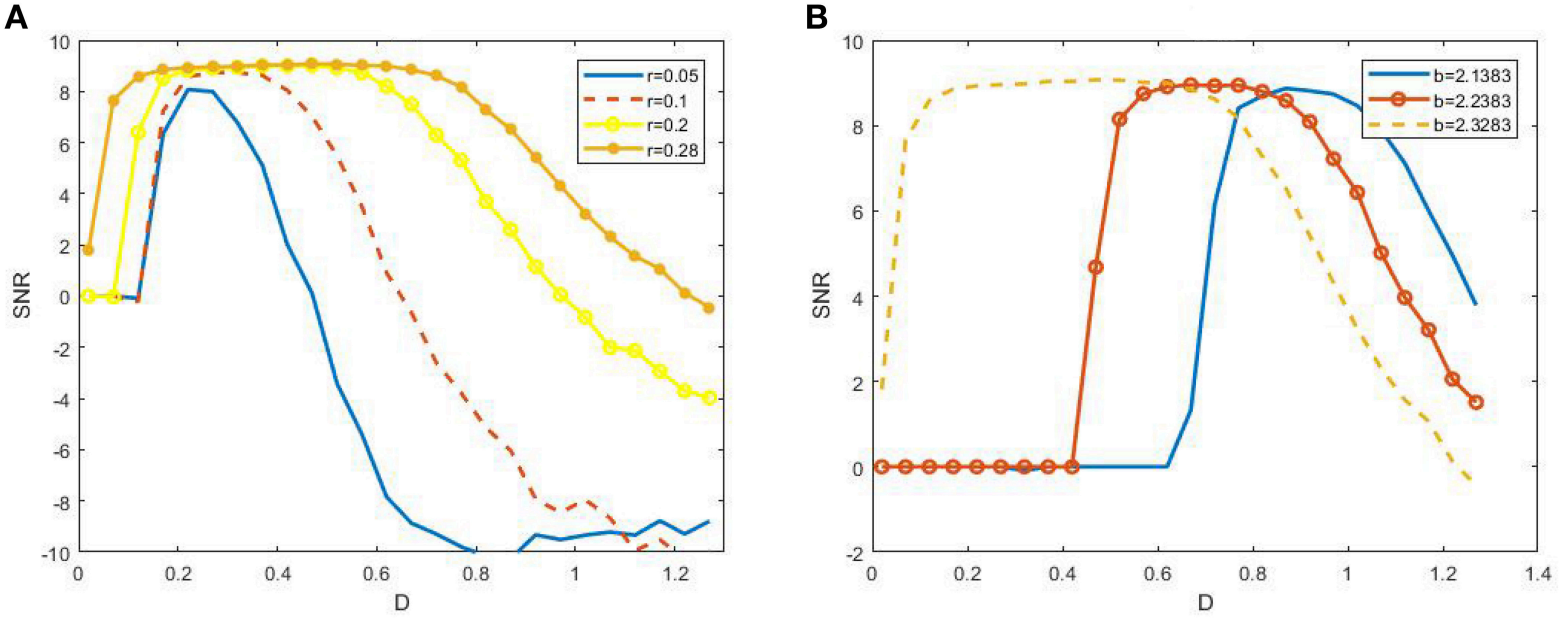
Dependence of signal to noise ratio on noisy intensity under different amplitude **(A)** or biased intensity of electromagnetic field **(B)** at ε = 0.005 and 2π*f* = 0.001. We fix *b* = 2.2328 in **(A)** and *r* = 0.28 in **(B)**. It is shown from **(A)** that the width of the resonant peak evidently enhances as the *r* increases, and from **(B)** that the resonant peak is dramatically shifted to larger noise intensity as *b* away from the critical bifurcation point (2.3383).

Many physical measure indexes are suitable for quantifying the phenomenon of SR, such as spectral amplification factor, mutual information, resident time distribution and signal-to-noise ratio (SNR), and all these indexes can reflect the beneficial role of noise from different viewpoints. Here, we choose the SNR defined by

$$SNR=10\log_{10}\frac{S(\omega )}{N(\omega )}$$

to characterize SR. Here *S*(ω) represents the height of the peak at the signal frequency and *N*(ω) is the power spectrum density of background noise. Figure 4 shows the dependence of SNR via noise intensity *D* under different signal amplitude *r* and biased intensity of electromagnetic field $\varphi_{ext}^{0}$, and these non-monotonic dependence curves exhibits the occurrence of SR in the model (3). This observation confirms that SR can occur in the presence of electromagnetic induction.

**Figure 5:**
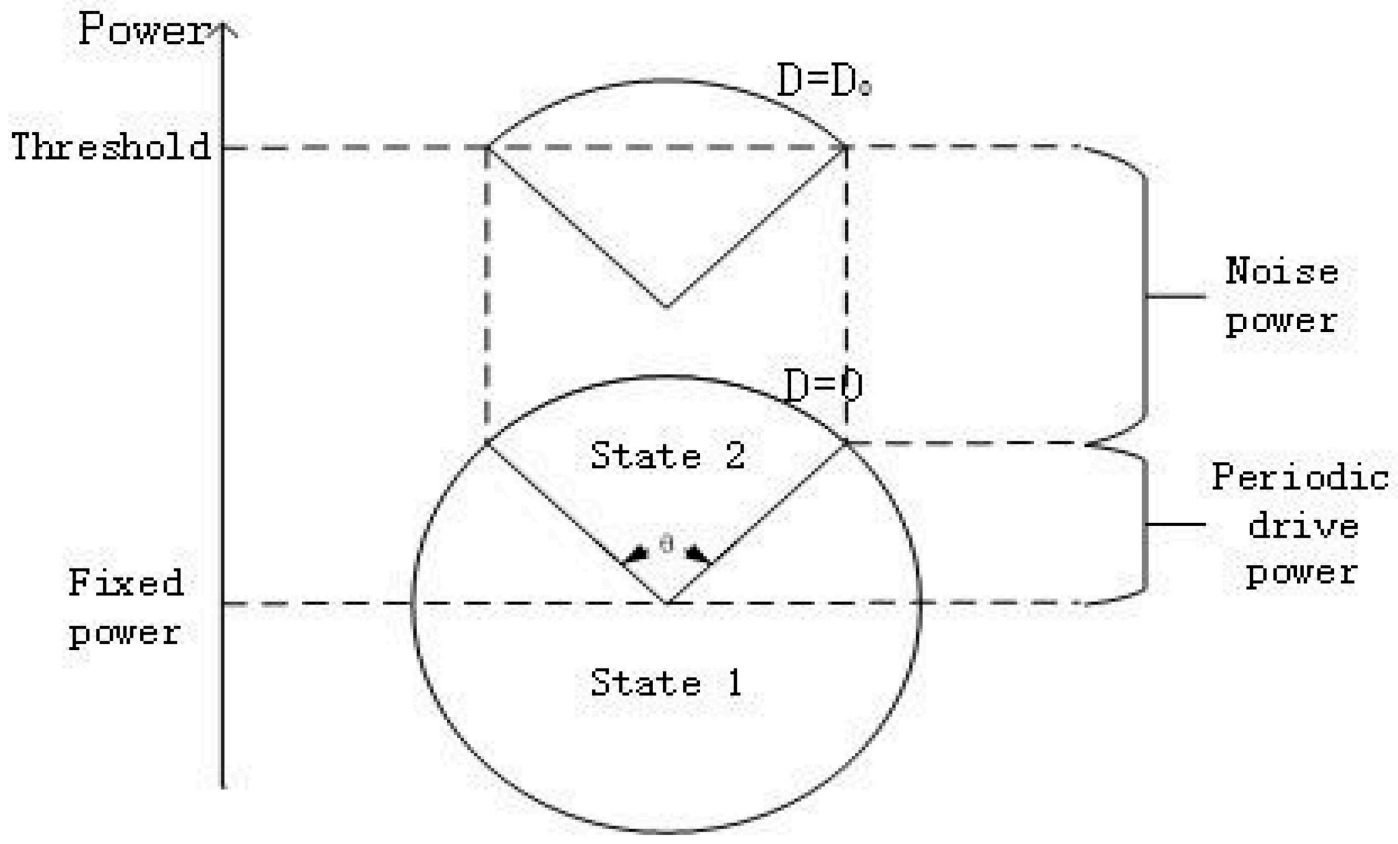
Qualitative analysis for stochastic resonance from the viewpoint of power. Every point on the circle represents the power offered only by the subthreshold signal ($r\sin\left(2\pi ft\right)+\varphi_{ext}^{0}$) to the system at different time. The height of center of the circle represents the fixed power offered by $\varphi_{ext}^{0}$. The power provided by the noise raises the height of the circle. From the schemata, there should be suitable noise level *D* = *D*_0_ such that the membrane potential transits between the state 1(resting state) to state 2(burst state) almost periodically, with a period corresponding to an arc with central angle θ(*D*).

Here let us explain why the phenomenon of SR occurs in the system (3) from the perspective of energy. As shown in Figure 5, the circle represents the power of the system offered by the subthreshold signal. If the power at some time is greater (less) than the fixed power as shown in the figure, it means the periodic signal is supplying (consuming) energy to the system. Obviously, when the noise is absent, the power from the subthreshold signal cannot drive the membrane potential to cross over the firing threshold, but with the help of the energy of a suitable noise, the membrane potential can cross the threshold over a time interval corresponding to an arc with a central angle θ. This to a certain extent is the dynamical mechanism underlying the occurrence of SR.

Now we turn to explain why the width of resonant peak becomes large as the signal amplitude enhances and why the resonant peak shifts to smaller noise intensity as the bifurcation parameter closes to the critical value, as observed in Figure 4. From Figure 5, it is clear that as the signal amplitude increases the radius of the circle becomes larger. This means that the power supplied by the periodic signal has more dominated capability in the interplay of noise and signal. As a result, the width of resonant peak for stronger signal will wider than that for the weaker signal. Similarity, when the bias intensity increases, the “Fixed power” in Figure 5 will be boosted and the required energy for the system to reach the threshold will become smaller. Hence, the resonant peak will appear at smaller noise intensity.

**Figure 6:**
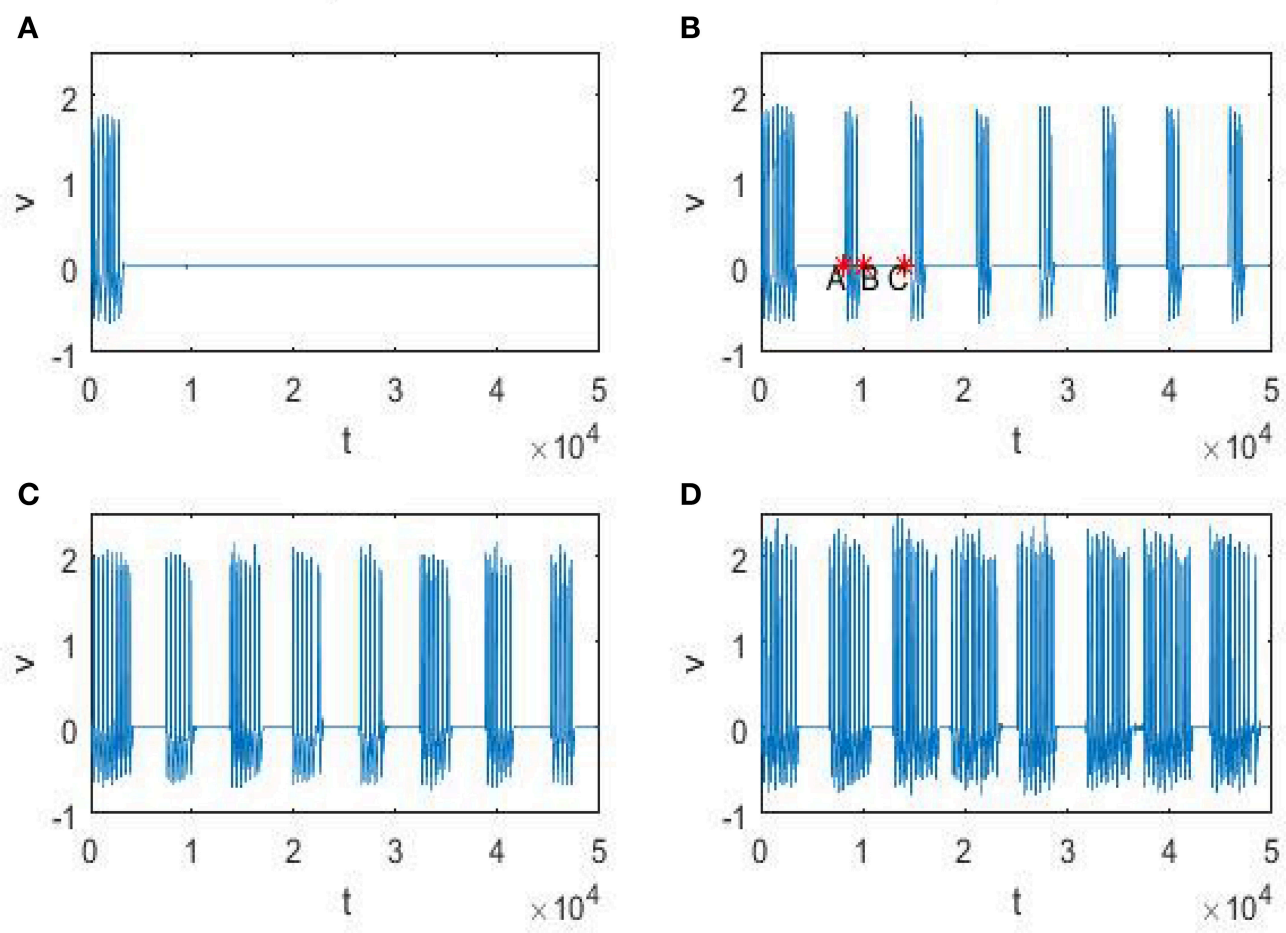
Time history of trans-membrane potential under different noise intensity *D*. The parameters ε = 0.005, *r* = 0.28, *f* = 0.001/2π, and $\varphi_{ext}^{0}=0.328$, and from **(A–D)**, the noise intensity *D* is 0, 0.25, 0.5, 0.75, respectively.

In order to explain the meaning of θ(*D*) in Figure 5, let us resort to the statistics of the time history of membrane potential (shown in Figure 6). It is easy to see that the firing pattern in Figure 6 belongs to bursting. Discarding the transient evolution, we can calculate the interburst intervals (IBIs), burst interval (BI) and resting interval (RI). Here the IBI is referred to as the time interval between adjacent bursts (Gritsun et al., 2011), and we define the BI as burst interval, namely the duration interval of a single bursting and the RI as the time interval from the end of the first burst to the beginning of the next one. For example, as shown in Figure 6B, the IBI, BI and RI correspond to *t*_*C*_ − *t*_*A*_, *t*_*B*_ − *t*_*A*_ and *t*_*C*_ − *t*_*B*_, respectively. Denoting *IBI*(*D*) = 〈*IBI*〉, *BI*(*D*) = 〈*BI*〉 and *RI*(*D*) = 〈*RI*〉, then θ(*D*) in Figure 5, proportional to time, can be calculated by

$$\theta (D)=2\pi \times \frac{BI(D)}{IBI(D)}=2\pi \times \frac{BI(D)}{BI(D)+RI(D)}.$$

**Figure 7:**
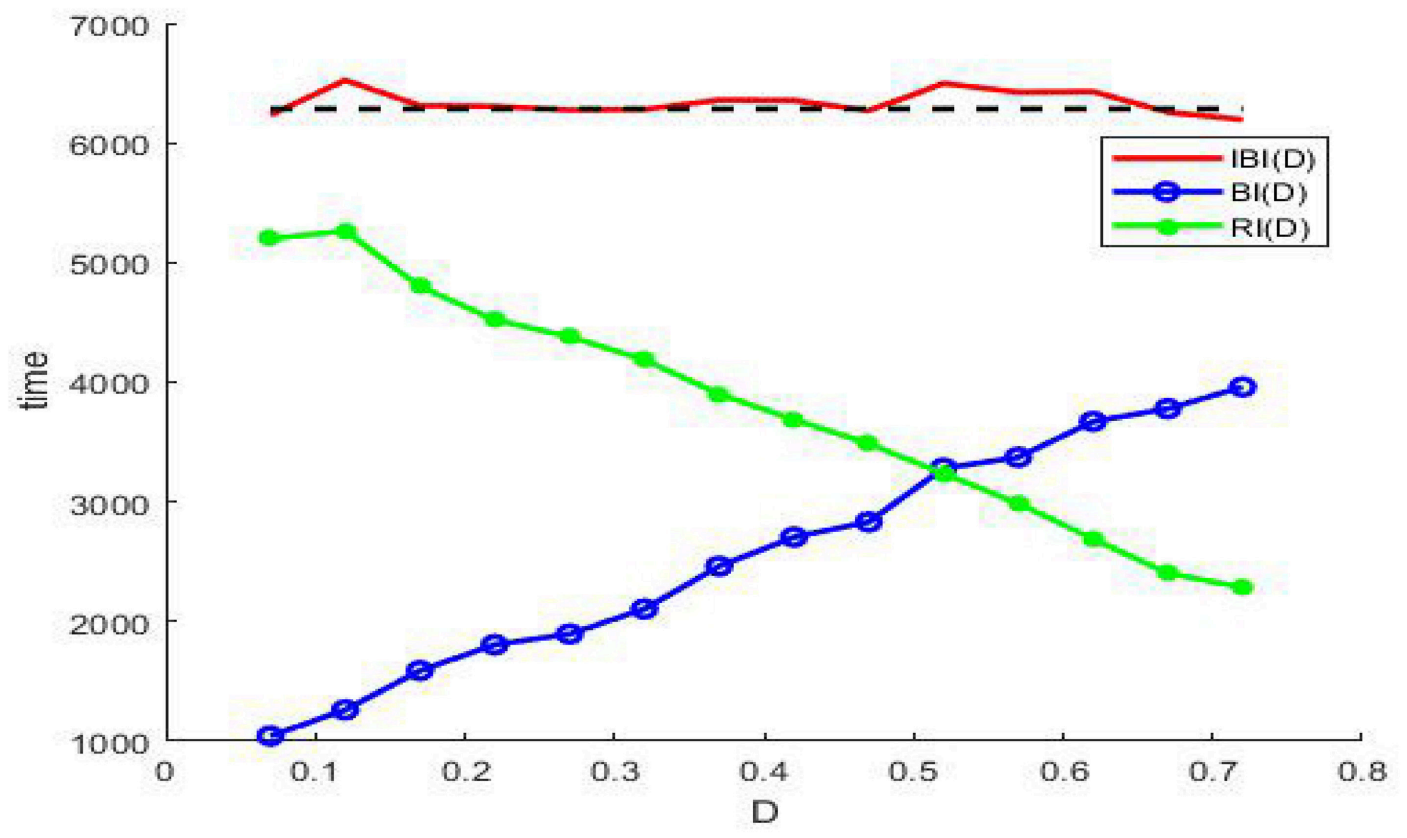
The evolution curves of *IBI*(*D*) (red solid), *BI*(*D*) (blue circle), and *RI*(*D*) (green dot) via noise intensity at ε = 0.005, *r* = 0.28, *f* = 0.001/2π, and $\varphi_{ext}^{0}=0.328$. Note that the *IBI*(*D*) cannot be defined when there is no noise, since the long time of the deterministic system (3) is static, but once the noise is induced and if the noise intensity falls within the range (0.07, 0.72) in the figure, the *IBI*(*D*) will fluctuate around the period of the sinusoidal drive (black dash), and this suggests that noise should be utilized by the model (3) in weak signal detecting. Here we emphasize that none of *IBI*(*D*)), *RI*(*D*), and *BI*(*D*) can be measured in absence of noise or in the case of too much noise, since the absence of noise leads the membrane potential to a static level, while too much noise causes the periodic bursting like patterns mix into one single noisy bursting.

By means of the evolution curves of these average intervals via the noise intensity *D* in Figure 7, θ(*D*) is less than π when the noise intensity is under the critical value at the intersection, and it is larger than π when the noise intensity is between this critical value and 0.7.

Noting that in the phenomenon of SR noise helps detect weak signal, so let us demonstrate how the weak subthreshold signal is detected in the system (3). It is clear that when the noise is absent, the membrane potential eventually stays at the resting level (Figure 6A), but as the noise intensity increases until attains a suitable range, the membrane potential will evolve according to an approximate periodic motion with fluctuations (Figures 6B–D), where the relevant approximate period is basically the signal period. Therefore, if we analyze the time history within this suitable noise range, the period of the weak signal will be identified. In fact, as shown in Figure 7, the mean IBI is nearly equal to the signal drive, although this quantity cannot be defined in the deterministic case.

**Figure 8:**
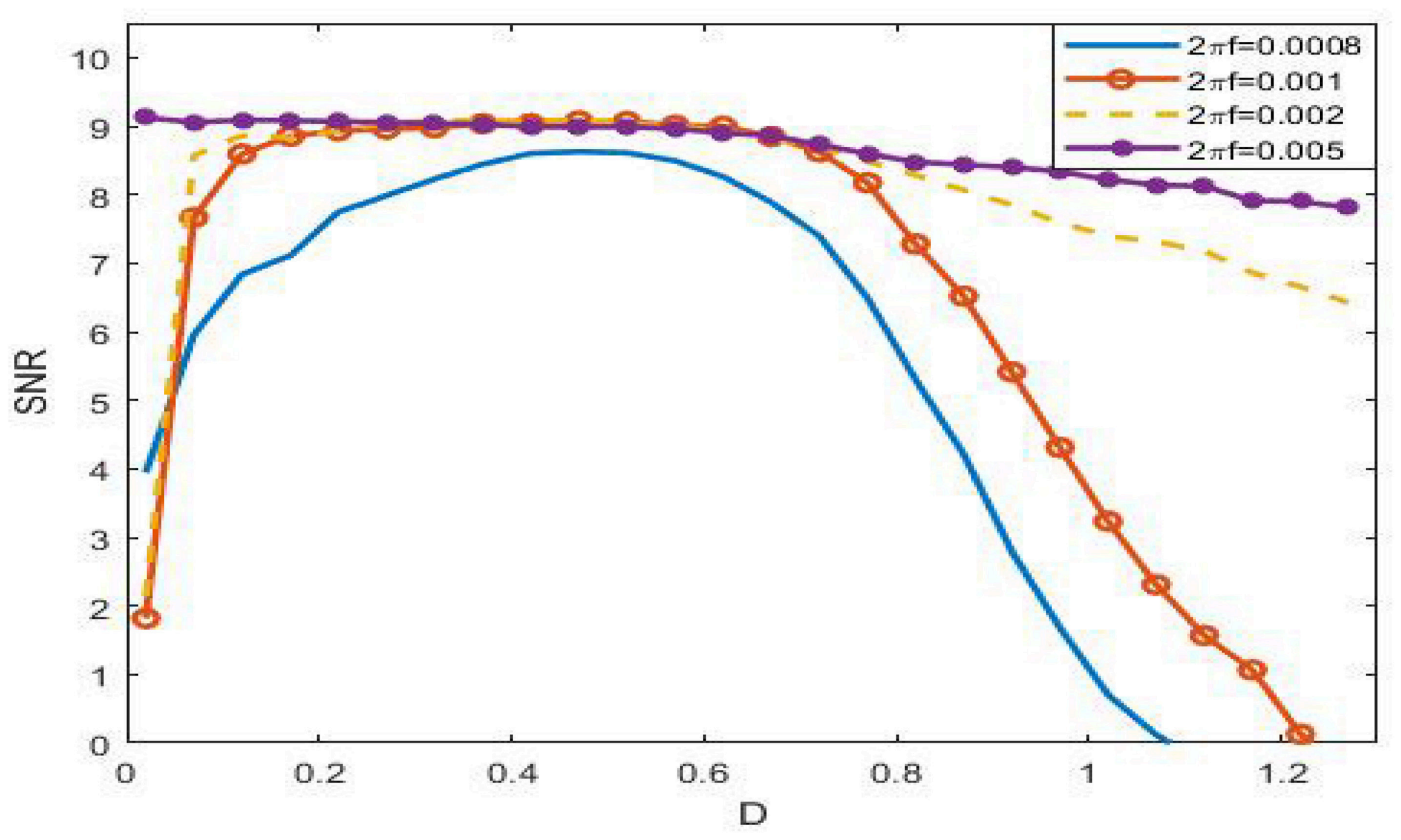
Dependence of signal to noise ratio on noisy intensity under different signal frequency at ε = 0.005, *r* = 0.28, and $\varphi_{ext}^{0}=0.328$. From the figure, it is clear that the SNR as a function of noise is monotonic, so SR can occur if 2π*f* ≤ 0.001, but if 2π*f* = 0.008, the function becomes monotonically decreasing, and as a result, there is no SR in this case. This figure tells that the phenomenon of SR has obvious frequency dependence, that is to say, the phenomenon can only occur within certain frequency range.

Viewing from the history of SR, the phenomenon was named after the term of “resonance” to some extent. In the symmetrical overdamped bistable system, the occurrence of SR is relating to a match relation between a half of the signal period and the mean first passage time, while in the underdamped oscillator system, there exists a coincidence between the driving frequency and the noise-tuned inherent frequency peak (Kang et al., 2003). These match relations suggest that there exists certain frequency interval such that the phenomenon of SR in the system (3) cannot occur if the signal frequency falls outside of this range. Figure 8 just verifies this point. For instance, when 2π*f* = 0.005, as the figure shows that the SNR as function of noise intensity monotonically decreases, which indicates no SR for the given signal amplitude.

## Conclusion and discussion

By taking the electromagnetic induction into account, an improved FHN model is proposed by means of the flux-controlled memristor. With the technique of linear stability analysis, the bifurcation diagram of the deterministic autonomous model is obtained and especially the subcritical Hopf bifurcation points are identified. For the noisy weak signal modulated system, we have observed the phenomenon of SR near the subcritical Hopf bifurcation point. From the viewpoint of energy we give an explanation of the occurring mechanism. By defining the several mean intervals relating to burst, we also discuss how to detect a weak signal based on the principle of SR in this model. Our investigations once again demonstrate that electromagnetic radiation can induce electrical activity in neurons. Moreover, our investigations suggest that the phenomenon of SR could be utilized by neurons in detecting weak signal in the presence of electromagnetic induction.

We would like to have some discussion on the phenomenon of SR disclosed in this investigation from weak signal detection. It is well known that neurons communicate with each other through action potential, and the timing of action potential trains often contains more significant information than their shape, thus it should be more inspiring to disclose the phenomenon of SR both from the noise dependence of the SNR and the statistical quantities of trains of interspike intervals (ISIs). Indeed, as pointed out in the introduction section, the experimental and theoretical investigations have suggested that noise helps in detecting or transmitting weak signal (Tanabe and Pakdaman, 2001; Hasegawa, 2004; Kang et al., 2005; Faisal et al., 2008; Mark et al., 2009; Jiao and Wang, 2010; Shao and Kang, 2014; Sun and Shi, 2014; Sun and Li, 2016), however, most of the existing investigations only disclosed SR by showing an optimal noise level which could make the system have a better SNR than other noise level, but failed to try to find the relation between the maximal SNR and the mean of ISIs. Different from these existing investigations, in this paper we find the relation of the maximal SNR and the evolution of the mean of IBIs (〈*IBI*〉), namely, the optimal noise level for the SNR is an interval and over the same interval the 〈*IBI*〉 is almost equal to the signal period. Although we are not sure whether this relation is universal in general neural systems, we infer it might be a predictable conclusion for SR occurring near subcritical Hopf bifurcation point. If this inference can also be confirmed in other neuron models, undoubtedly it will provide a biologically feasible scheme for weak signal detection.

## Author contributions

Y-XF designed the model, did most of the numerical calculations and finished the initial draft. Y-MK guided in elementary methods about linear stability analysis and stochastic resonance, and she rewrote the whole presentation. YX drew Figure 1 with the software of xppaut.

### Conflict of interest statement

The authors declare that the research was conducted in the absence of any commercial or financial relationships that could be construed as a potential conflict of interest.
